# Balneotherapy in Fibromyalgia Syndrome: protocol of “FIBROTHERM”, a prospective multi-center, two-cohort observational study

**DOI:** 10.1007/s00484-025-02876-w

**Published:** 2025-03-05

**Authors:** Alarico Ariani, Giorgio Bedogni, Giovanni Biasi, Franco Cozzi, Sabrina Formisano, Roberto Gorla, Serena Guiducci, Maria Chiara Maccarone, Stefano Masiero, Simona Montalbano, Maurizio Muratore, Francesca Nacci, Eugenio Quarta, Gianluca Regazzo, Francesca Regola, Riccardo Terribili, Enrico Tirri, Rosella Tirri, Marco Vitale, Antonella Fioravanti

**Affiliations:** 1 Internal Medicine and Rheumatology Unit, University Hospital of Parma, Parma, Italy; 2 Department of Medical and Surgical Sciences, Alma Mater Studiorum University of Bologna , Bologna, Italy; 3 Department of Primary Health Care, AUSL Romagna, Ravenna, Italy; 4 Rheumatology Unit-Azienda Ospedaliera-Senese, Siena, Italy; 5 Villa Salus Hospital, Venice-Mestre, Italy; 6 Unit of Rheumatology, Department of Precision Medicine, University of Campania L. Vanvitelli, Caserta, Italy; 7 Unit of Rheumatology and Clinical Immunology, ASST Spedali Civili, Brescia, Italy; 8 Division of Rheumatology, Department of Clinical and Experimental Medicine, University of Florence, Florence, Italy; 9 Department of Neuroscience, Physical Medicine and Rehabilitation Unit, University of Padua, Padua, Italy; 10 Department of Medicine, Rheumatology Unit, Ospedale del Mare, Naples, Italy; 11 Rheumatology Unit, “Vito Fazzi”, Lecce, Italy; 12 Faculty of Medicine & Surgery, Vita-Salute University – San Raffaele Hospital, Milan, Italy; 13 Foundation for Scientific Research in Balneology (Forst), Rome, Italy; 14 World Hydrothermal Organization (Omth), Levico Terme, Italy

## Abstract

Balneotherapy (BT) is considered an effective, non-pharmacological approach, in the multimodal treatment of the Primary Fibromyalgia Syndrome (FS). However, the evidence of efficacy and tolerability of BT in FS is still limited. This is a prospective multi-center two-cohort observational study. The main aim will be the comparison of the Minimal Clinically Important Difference (according to Fibromyalgia Impact Questionnaire—FIQ) achievement in FS patients treated with BT vs standard care. Secondary objectives will be to assess: a) BT impact on pain, quality of life, anxiety and depression; b) the persistence of benefits in six weeks c) BT safety profile. All FS patients with a stable treatment in the past 3 months and a moderate to severe disease (FIQ score ≥ 39) will be enrolled after providing written informed consent. Patients will be divided into two Cohort: a) BT Cohort (i.e., BT in addition to standard care)—BTC; b) Control Cohort (i.e., only standard care)—SCC. There will be three assessments: baseline, two and six weeks (i.e., one month after BT end in BTC). At each of them the subject will fill in the following questionnaires: FIQ, VAS pain, Short Form Health Survey 16, State-Trait Anxiety Inventory and Center for Epidemiological Studies Depression Scale. We expect to observe a more relevant improvement of disease activity in BTC than in SCC. The positive effect may extend even to pain, quality of life, anxiety and depression. The short- and medium-term effects are likely to be similar, without any significant warning in terms of tolerability. Collected data, deriving from a large sample of patients, will provide a new insight of BT role in moderate to severe FS treatment. In particular, it will be possible to quantify the short and medium-term BT impact on disease activity and secondary symptoms related to FS.

## Introduction

Fibromyalgia Syndrome (FS) is a long-lasting condition, characterized by the ongoing presence of widespread musculoskeletal pain, fatigue, sleep disruptions, headaches, irritable bowel syndrome, as well as anxiety and depression (Mease 2005; Sarzi-Puttini et al. 2020). It affects between 1 and 5% of the general population, with women being six times more likely to be diagnosed than men (Giorgi et al. 2023). FS significantly impacts patients’ quality of life (Häuser et al. 2018). Pain and functional impairments often result in work absenteeism, frequent medical consultations, and the pursuit of various conventional or alternative treatments, leading to substantial direct and indirect healthcare costs (Spaeth 2009; Vervoort et al. 2016).

Despite advancements in understanding its pathophysiology, there are still no specific pharmacological treatments for FS. Current international guidelines recommend a multidisciplinary and multimodal approach, combining both pharmacological and non-pharmacological therapies (Macfarlane et al. 2016).

Balneotherapy (BT) includes a wide array of therapeutic methods such as hydrotherapy, bathing in mineral-rich water, mud therapy, or utilizing natural remedies, typically performed in health spas (Gutenbrunner et al. 2010; Vaccarezza and Vitale 2010; Fioravanti et al. 2017; Antonelli et al. 2021). In 2002, the World Health Organization (WHO) categorized BT within traditional or complementary medicine, acknowledging its broad therapeutic applications (WHO 2002). Furthermore, the European League Against Rheumatism (EULAR) and the Italian Society of Rheumatology (SIR) endorse BT as a valid treatment option within the multimodal management of FS (Macfarlane et al. 2016; Ariani et al. 2021).

Several meta-analyses, systematic reviews, randomized controlled trials, and observational studies confirm the efficacy and tolerability of BT in managing FS (Fioravanti et al. 2007; Naumann and Sadaghiani 2014; Cao et al. 2021; Maindet et al. 2021; Ducamp et al. 2022; Colas et al. 2024; García-López et al. 2024). However, there are limited studies that focus on the effects of BT on pain, functional symptoms, health-related quality of life, anxiety, and depression in a large group of patients with moderate to severe FS. We hypothesize that combining BT with standard treatment is both safe and more effective than standard treatment alone in managing FS patients.

The primary aim of this study will be to evaluate whether BT increases the proportion of patients achieving the Minimal Clinically Important Difference (MCID) as measured by the Fibromyalgia Impact Questionnaire (FIQ), compared to standard care. Secondary aims will be a) the assessment of BT additional effects on pain, quality of life, anxiety and depression, b) the persistence of clinical efficacy over a six-week follow-up, c) the tolerability of BT by recording all adverse events, their severity and possible relationship with the therapy.

## Methods

### Study design and ethics

FIBROTHERM is a prospective multi-center, two-cohort observational study. The enrollment period will take place from April 2023 to November 2024 (deadline for the enrollment of the last patient) by the centers participating in the study. From June 2025, the data will be analyzed and then, disseminated. The estimated total duration of the study will be 26 months.

The study will be conducted in accordance with the “Declaration of Helsinki (1964) and following amendments. All eligible patients will provide a written informed consent, and this will be recorded in the appropriate outpatient file. The study protocol was approved by the Ethics Committee of the Area Vasta Sud Est-Toscana (Italy) on March 2023; the same document was submitted and approved by local Ethics Committees of the other participating centers to ensure that all local regulatory requirements were met. Furthermore, the study was registered on http://www.clinicaltrials.gov: NCT05801497.

### Participants

#### Inclusion criteria

Patients of both sexes meeting the 2016 American College of Rheumatology (ACR) criteria for primary FS (Wolfe et al. 2016) and aged between 18 and 75 years will be eligible for this study. Indeed, for this study we will include patients with a FIQ score ≥ 39 (i.e. moderate to severe disease) and receiving stable pharmacological and non-pharmacological treatment over the previous three months.

#### Exclusion criteria

Exclusion criteria will include: 1) enrollment in other research protocols in the 6 months before the study begins; 2) BT treatment in the last 6 months; 3) intra- or extra-articular steroid injections in the previous 3 months; 4) pregnancy or breastfeeding; 5) presence of cognitive disorders, psychiatric illnesses and/or drug addictions (including alcohol) that can hinder the administration of the questionnaires; 6) absolute or relative contraindications to BT as reported in the literature (Fioravanti and Marcolongo 2000).

### Settings of the enrollment and where the data were collected

Patients with primary FS will be enrolled in the Rheumatology or Rehabilitation Units of public general Hospitals and public University Hospitals. Currently, nine centers, located throughout Italy, have joined the FIBROTHERM study. The same centers will be responsible for clinical data collection.

### Interventions and study arms

This study consists of two parallel cohorts:

- BT Cohort (BTC): Patients will receive a cycle of bath or of mud-bath therapy once daily for 12 days in addition to their usual treatments for FS (see below). The thermal treatment cycle will include one session per day for a total of two weeks, with a break between the fifth and seventh day to prevent the onset of a thermal crisis. This protocol aligns with current practices and is endorsed by the Italian National Health Service which covers a cycle of a 12-day treatments, once a year for patients with rheumatic diseases such as FS. The mineral thermal baths will be carried out in a single tub or in a pool, with a duration of 10 min at a temperature of 37–38° C. The thermal mud will be applied to the skin according to standardized methods, with a duration of 15–20 min, at temperatures ranging between 40–45° C. A rest period of about 20–30 min will follow BT treatment. The whole treatment duration will last 1 h, approximately. Only muds and baths belonging to the classes of sulfurous, bicarbonate, chloride-sodium, sulphate and arsenical-ferruginous waters—according to the classification of Marotta and Sica (1933), will be allowed.
- Control Cohort (SCC): This group will include patients who will refuse the BT for personal reasons. Patients will continue their established routinely care for FS.

The usual care treatment will correspond to pharmacological and non-pharmacological therapies as reported in EULAR guidelines (Macfarlane et al. 2016). The intervention program must have already been stabilized for at least 3 months before the start of the study. During the study period, the patient can modify the therapy if necessary; this change will be reported.

### Outcomes

#### Primary outcome

The primary outcome of the study will be the percentage of patients with a minimal clinically important difference (MCID), defined as a decrease of more than 14% in their FIQ-Total score, at 15 days (Bennett et al. 2009; García-López et al. 2024).

The FIQ is a self-administered questionnaire evaluating the impact of the disease in daily activities; the score ranges from 0 to 100, and higher scores indicate a greater impact of the disease (Table 1). A score lower than 39 is considered a mild impact, 39 to 59 is considered moderate, and greater than 59 is considered severe (Burckhardt et al. 1991). In the present study, we will use the Italian translated validated version (Sarzi-Puttini et al. 2003).

**Table 1:** Description of tests which will be administrated

Test	Description
Fibromyalgia Impact Questionnaire (FIQ)	FIQ is a self-administered instrument composed of 10 items that measure fibromyalgia patient status, progress and outcomes. Each question is rated on a 4-point Likert-type scale
Visual Analogue Scale (VAS)	Unidimensional measure of patients’ pain intensity, used to record pain progression and compare pain severity between patients
Widespread Pain Index (WPI)	19-point checklist that assesses the presence of pain or tenderness in 19 specific areas of the body; each affected area receives one point
Short-Form Health Survey (SF-12)	Self-reported outcome measure assessing the impact of health on an individual's everyday life. It is often used as a quality of life measure
State-Trait Anxiety Inventory (STAI)	STAI is a test that measures two types of anxiety – state anxiety and trait anxiety. It consists of 40 self-report items on a 4-point Likert scale, with higher scores representing higher levels of anxiety
Centre for Epidemiologic Studies Depression Scale (CES-D)	Brief self-report questionnaire developed to measure depressive symptoms severity. The CES-D consists of 20 questions that ask about various symptoms of depression as they have occurred in the past week

#### Secondary outcomes

##### Pain

Pain intensity will be evaluated by a Visual Analogue Scale (VAS), a 10-cm long line with the value 0 on the left indicating “no pain” and the value 10 on the right indicating the “worst imaginable pain” (Scott and Huskisson 1976).

Furthermore, we will consider as a secondary outcome, the Widespread Pain Index (WPI) that is a measure of the number of painful areas (score range = 0–19) on the patient’s body over the last week before the assessment (Wolfe et al. 2010).

##### Quality of life

The quality of life will be assessed by the Short-Form Health Survey (SF-12), a short version of SF-36 that is a widely used measure of health and well-being, validated in multiple countries, including Italy (Ware et al. 1996; Apolone and Mosconi 1998).

SF-12 includes two main domains: the physical component score (PCS) and the mental component score (MCS), and eight scales for assessing eight dimensions: physical functioning, physical role, social role, emotional role, bodily pain, general health, vitality, and mental health. Scores range from 0 to 100 where 0 indicates the worst condition and 100 indicates the best possible condition.

##### Anxiety

The anxiety will be evaluated by the State-Trait Anxiety Inventory (STAI): this a self-administered test with two independent scales (STAI T-Anxiety Scale or Form X-2 and STAI S-Anxiety Scale or Form X-1); each one includes 20 items. A high score on the STAI corresponds to a high level of anxiety symptoms. For the present study, we will use the Italian validated STAI (Spielberger et al. 1983; Lazzari and Pancheri 1980).

##### Depression

The assessment of depression will be performed by the Italian validated version of Centre for Epidemiological Studies Depression Scale (CES-D) (Radloff 1977; Fava 1983). It is a self-administered 20 items questionnaire evaluating the frequency of depression symptoms in the previous week; scores range from 0 to 60, with high scores indicating greater depressive symptoms. Scores of 16 or above are considered at risk for clinical depression.

##### Tolerability

All adverse events, whether reported spontaneously by the patients or observed by the physician, will be reported in the personal record noting the severity. The adverse event will be considered related to the treatment if it appears after the start of the therapy or if it was known as a BT side effect. In addition, side effects related to drug therapy will be monitored and reported as part of the study.

### Follow up

**Fig. 1:**
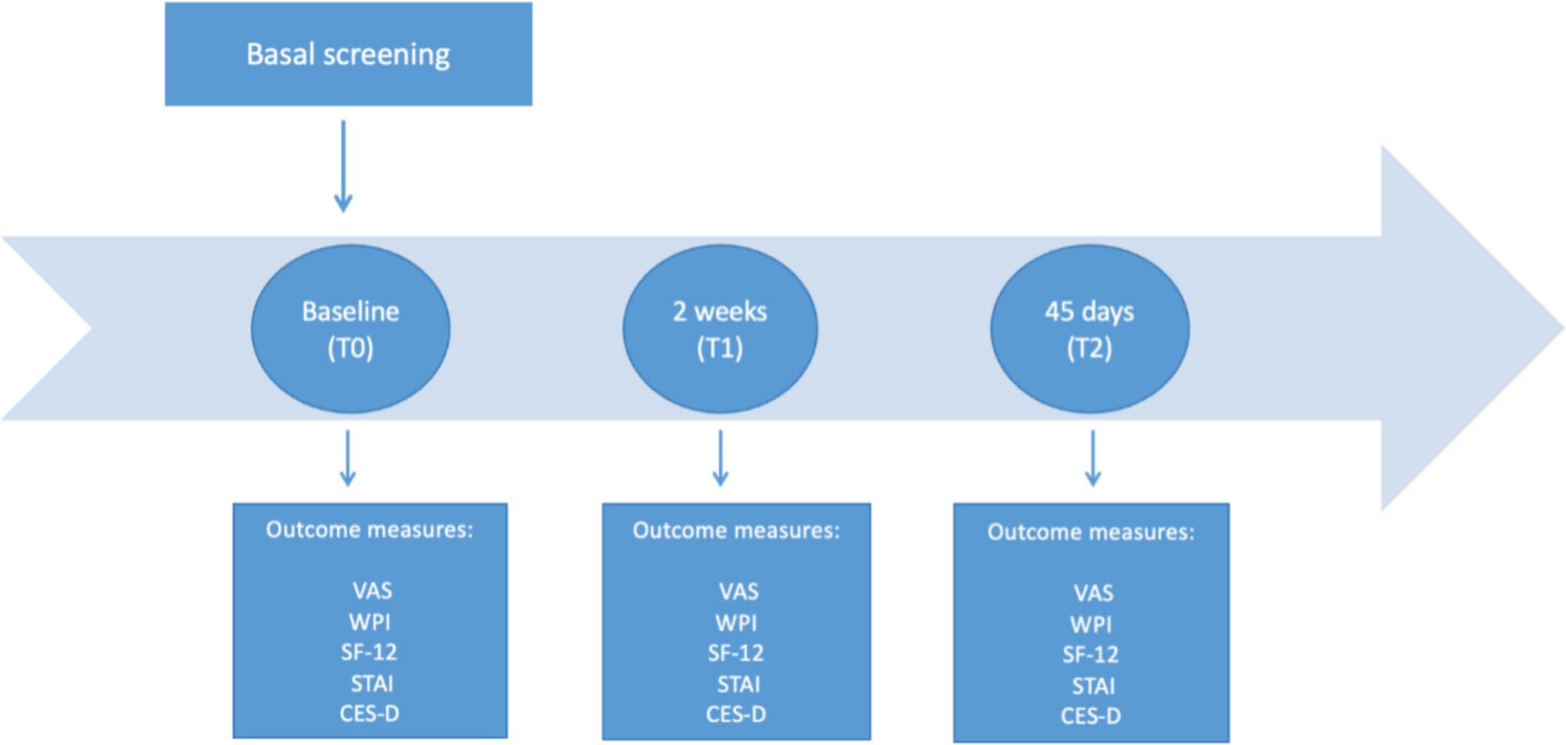
FIBROTHERM timeline

Following enrollment, each patient will be examined for 3 consecutive times; at baseline (T0), after 2 weeks (T1), and after 45 days (T2) (one month after the end of the cycle of the therapy for the BT Cohort) (Fig. 1). T0 and T1 visits will be carried out by the physician in the enrollment setting. The T2 visit will be carried-out by phone. The study will be considered concluded when all follow-up data for each patient will be collected.

### Sample size

The primary outcome is the modification of the FIQ total score, whose minimum clinically relevant difference is 14% as compared to its initial value (Burckhardt et al. 1991). We expect a decrease in the FIQ total score of at least 14% in the 30% of the control cohort and 45% in the BT cohort. A number of 217 patients per group allows estimating an absolute difference of 15% (45—30%) in the incidence of the primary outcome between the two cohorts at an alpha level = 0.05 and with a power of 90% (Pearson’s chi-squared test). Assuming a loss to follow-up of 20%, 260 (217 + 43) patients will be enrolled per group, for a total of 520 patients.

### Statistical analysis plan

#### Primary outcome

The primary outcome of the study will be the difference in the proportion of patients with reduction in FIQ-Total score > 14% in the BT vs. the control cohort. As described above, Pearson’s chi-square test will be used to evaluate the statistical significance of this difference, considered clinically relevant if ≥ 15%, at an alpha = 0.05 level and with a power of 90%. Subsequently, a binomial regression model (BRM) will be used to quantify the size of this difference and its precision (95% confidence interval) (Long and Freese 2006). The response variable of the BRM will be the proportion of subjects with a reduction in the FIQ-Total score > 14% at 15 days, and the predictor will be the cohort (discrete: 0 = control cohort; 1 = BT cohort). The effect size estimated by the BRM will be expressed both as absolute and relative risk change. Marginal estimates with 95% confidence intervals of the percentages of patients with reduction in FIQ-Total score > 14% in the two cohorts will be calculated.

In the BT cohort, we will also use a BRM having as a response variable, the proportion of subjects with a reduction in the FIQ-Total score > 14% and time as predictor (discrete; 0 = 15 days; 1 = 45 days) to verify the stability of the change in the FIQ-Total score at 30 days. A Bonferroni correction will be used to correct the 95% confidence intervals and associated p-values. The univariable BRM described above for the evaluation of the primary outcome between the BT and control cohorts will subsequently be expanded to a multivariable model by adding predictors (= potential confounders) determined preliminarily with the clinicians participating in the study. An univariable selection of the predictors will not be made, but they will be progressively added to the model to verify the effect on the relative risk estimated by the model itself (Greenland and Pearce 2015). Interactions initially determined or suggested by the multivariable analysis may also be included among the predictors. It is possible, and indeed expected, that the BRM will have to be replaced by a Poisson regression model (PRM) with robust confidence intervals, to overcome the well-known problems of fitting a BRM with multiple predictors (Zou 2004; Lumley et al. 2006). At this stage, we will only model the relative risk because, being this a cohort study, the relative risk measure is expected to be more transferable than the absolute risk.

#### Secondary outcomes

The secondary outcomes of the study will be the differences in VAS, SF-12, STAI, and CES-D between the BT and control cohorts. These differences will be evaluated using a random effects linear regression model (RE-LRM) (Rabe-Hesketh and Skrondal 2021) 39. The response variable of such models will be the (continuous) score obtained from the scale and the predictors will be: 1) time (discrete; 0 = baseline; 1 = 15 days), 2) the cohort (discrete; 0 = control cohort and 1 = BT cohort), and 3) a time*cohort (discrete*discrete) interaction, with the random effect assigned to the patient. A Bonferroni correction will be used to correct the 95% confidence intervals and associated p-values. As already described for the BRM used for the evaluation of the primary outcome, also the RE-LRM will be expanded to a multivariable model by adding predictors (= potential confounders) determined preliminarily with the clinicians participating in the study. Interactions initially determined or suggested by the multivariable analysis may also be included among the predictors. An univariable selection of the predictors will not be carried out, but predictors will be progressively added to the model to verify the effect on the estimate of the difference in the scores for the BT and control cohort (Greenland and Pearce 2015).

#### Statistical techniques common to the analysis of primary and secondary outcomes

We will use multivariable fractional polynomials to test the linearity of the predictor-log risk association in the case of BRM/PRM and that of the predictor-score association in the case of RE-LRM (Royston and Sauerbrei 2008). In the case of missing data, provided that a missing at random (MAR) mechanism is justifiable, we will use multiple imputation for chained equations (MICE) to obtain plausible values of the missing variables from those available (White et al. 2011; van Buuren 2018). To this end, we will use at least 100 multiple imputation datasets. (Note that RE-LRM models are still reliable in the presence of MAR-type missingness.) The statistical analysis will be performed using Stata 18.5 (Stata Corporation, College Station, TX, USA) and R 4.4.1 (R Foundation for Statistical Computing, Vienna, Austria, EU).

## Discussion

FS is highly prevalent in the general population, with its incidence having risen dramatically in the past decade (Giorgi et al. 2023). The condition is primarily characterized by chronic, widespread pain and is often accompanied by additional symptoms such as anxiety, depression, sleep disturbances, and cognitive issues, all of which significantly affect the patients’ quality of life (Mease 2005; Sarzi-Puttini et al. 2020).

Effective management of FS requires a multidisciplinary and multimodal approach, combining pharmacological and non-pharmacological treatments (Macfarlane et al. 2016; Ariani et al. 2021). Non-pharmacological therapies are particularly recommended as the first line of treatment (Kundakci et al. 2022; Jurado-Priego et al. 2024). Among these, BT is recognized as a valuable complementary treatment, with numerous meta-analyses, systematic reviews, and randomized controlled trials supporting its efficacy, tolerability, and safety in FS management (Fioravanti et al. 2007, 2018; Naumann and Sadaghiani 2014; Cao et al. 2021; Maindet et al. 2021; Ducamp et al. 2022; Colas et al. 2024; García-López et al. 2024).

The goal of this study will be to assess the effects of BT on pain, function, quality of life, anxiety, and depression in a large cohort of patients with moderate to severe FS, through a prospective, multi-center observational study. We plan to enroll patients with primary FS who have FIQ score of 39 or higher and have had stable treatment for at least three months.

BT has been shown to provide significant benefits in managing FS, addressing both physical and psychological symptoms of the disease. Current scientific literature and our previous studies indicate that BT can lead to improvements in pain, physical function, quality of life, and anxious-depressive symptoms, with effects lasting beyond the immediate post-treatment period (Fioravanti et al. 2007, 2018; Paoloni et al. 2017; Maccarone et al. 2023). Therefore, we hypothesize significant improvements both immediately following treatment and one month after the treatment. The therapeutic effects of BT are multifaceted (Cheleschi et al. 2021). Hot stimuli have analgesic effects and reduce muscle tone; BT increases the secretion of β-endorphin, corticotrophin, cortisol, growth hormone, prolactin and influences the serotonin and dopamine function helping to improve the characteristic symptoms and quality of life of FS patients (Cheleschi et al. 2021; Gálvez et al. 2024; Antonelli et al. 2024a, b). BT treatments decrease circulating levels of Prostaglandin E2 (PGE2), Leukotriene B4 (LTB4) and Interleukin-1β (IL-1β), important mediators of inflammation and pain in this pathological condition (Ardiç et al. 2007). Finally, BT is able to reduce oxidative stress in patients suffering from FS (Çetinkaya et al. 2020).

Furthermore, combining BT with physical therapy or other standard treatments has been shown to produce superior outcomes compared to conventional therapies alone, especially in terms of long-term pain relief, respiratory function, and overall disability (Tognolo et al. 2022). Therefore, we also expect that the combination of BT with standard therapy (both pharmacological and non-pharmacological) will demonstrate superior outcomes compared to standard therapy alone.

BT also offers significant mental health benefits, particularly in reducing anxiety and depressive symptoms common in FS. Its anti-inflammatory effects may influence brain chemistry, as inflammation is linked to mood disorders like depression (Fioravanti et al. 2024, Koroglu and Yıldız 2024). Notably, IL-6, an inflammatory marker, is associated with sleep disturbances, and BT’s ability to regulate IL-6 could be a key mechanism behind its positive effects on sleep quality. In addition, warm, mineral-rich water promotes relaxation and reduces stress, contributing to better sleep patterns (Antonelli et al. 2024a, 2024b). This restorative sleep further enhances the benefits of BT by reducing fatigue and improving psychological resilience, ultimately leading to better overall well-being (Moini Jazani et al. 2023; Manica et al. 2024).

Furthermore, we expect to confirm a good tolerability of BT in a large sample of patients with moderate-severe FS.

In conclusion, the favorable effect of BT extended to several domains, including quality of life, anxiety and depression, could be particularly useful in the multimodal treatment of this disease.

A notable strength of this study is its large sample size and the involvement of nine specialized public facilities across Italy, which ensures a comprehensive representation of the national context.

Moreover, the results of this study have the potential to provide valuable insights that could help shape best practices for the clinical management of FS. Additionally, it may offer new evidence to further validate the role of BT as part of the complex approach to treating FS.

Nevertheless, there are some limitations to consider. Firstly, the observational nature of the study is a key limitation. Secondly, there will be no standardization of the BT treatments, as different mineral waters and specific protocols will be employed at each center, which is consistent with a real-world study design. Similarly, the routine care for FS, including both pharmacological and non-pharmacological treatments, will not be standardized but will follow the recommendations provided by the EULAR guidelines (Macfarlane et al. 2016).

## Data Availability

The data collected and analyzed for the current study will be available from the corresponding author on reasonable request.

## Code availability

NA.
